# Methicillin-Resistant *Staphylococcus aureus* (MRSA) Infection of Diabetic Foot Ulcers at a Tertiary Care Hospital in Accra, Ghana

**DOI:** 10.3390/pathogens10080937

**Published:** 2021-07-24

**Authors:** Ramzy B. Anafo, Yacoba Atiase, Nicholas T. K. D. Dayie, Fleischer C. N. Kotey, Patience B. Tetteh-Quarcoo, Samuel Duodu, Mary-Magdalene Osei, Khalid J. Alzahrani, Eric S. Donkor

**Affiliations:** 1 Department of Medical Microbiology, University of Ghana Medical School, Accra P.O. Box KB 4236, Ghana; rambugri201248@gmail.com (R.B.A.); ntkddayie@ug.edu.gh (N.T.K.D.D.); fcnkotey@flerholiferesearch.com (F.C.N.K.); pbtetteh-quarcoo@ug.edu.gh (P.B.T.-Q.); mmosei@ug.edu.gh (M.-M.O.); 2 Department of Medicine, University of Ghana Medical School, Accra P.O. Box KB 4236, Ghana; yacobat@gmail.com; 3 FleRhoLife Research Consult, Accra P.O. Box TS 853, Ghana; 4 Department of Biochemistry, Cell and Molecular Biology, University of Ghana, Accra P.O. Box LG 54, Ghana; saduodu@ug.edu.gh; 5 West African Centre for Cell Biology of Infectious Pathogens, University of Ghana, Accra P.O. Box LG 54, Ghana; 6 Department of Clinical Laboratory Sciences, College of Applied Medical Sciences, Taif University, Taif P.O. Box 11099, Saudi Arabia; Ak.jamaan@tu.edu.sa

**Keywords:** multidrug-resistant, *Staphylococcus aureus*, MRSA, infection, diabetic foot ulcer

## Abstract

Aim: This study investigated the spectrum of bacteria infecting the ulcers of individuals with diabetes at the Korle Bu Teaching Hospital in Accra, Ghana, focusing on *Staphylococcus aureus* (*S. aureus*) and methicillin-resistant *S. aureus* (MRSA), with respect to their prevalence, factors predisposing to their infection of the ulcers, and antimicrobial resistance patterns. Methodology: This cross-sectional study was conducted at The Ulcer Clinic, Department of Surgery, Korle Bu Teaching Hospital, involving 100 diabetic foot ulcer patients. The ulcer of each study participant was swabbed and cultured bacteriologically, following standard procedures. Antimicrobial susceptibility testing was done for all *S. aureus* isolated, using the Kirby-Bauer method. Results: In total, 96% of the participants had their ulcers infected—32.3% (*n* = 31) of these had their ulcers infected with one bacterium, 47.9% (*n* = 46) with two bacteria, 18.8% (*n* = 18) with three bacteria, and 1.0% (*n* = 1) with four bacteria. The prevalence of *S. aureus* and MRSA were 19% and 6%, respectively. The distribution of the other bacteria was as follows: coagulase-negative Staphylococci (CoNS) (54%), *Escherichia coli* (24%), *Pseudomonas* spp. (19%), *Citrobacter koseri* and *Morganella morgana* (12% each), *Klebsiella oxytoca* (11%), *Proteus vulgaris* (8%), *Enterococcus* spp. (6%), *Klebsiella pneumoniae* (5%), *Proteus mirabilis* and *Enterobacter* spp. (4%), *Klebsiella* spp. (2%), and *Streptococcus* spp. (1%). The resistance rates of *S. aureus* decreased across penicillin (100%, *n* = 19), tetracycline (47.4%, *n* = 9), cotrimoxazole (42.1%, *n* = 8), cefoxitin (31.6%, *n* = 6), erythromycin and clindamycin (26.3% each, *n* = 5), norfloxacin and gentamicin (15.8% each, *n* = 3), rifampicin (10.5%, *n* = 2), linezolid (5.3%, *n* = 1), and fusidic acid (0.0%, *n* = 0). The proportion of multidrug resistance was 47.4% (*n* = 9). Except for foot ulcer infection with coagulase-negative Staphylococci, which was protective of *S. aureus* infection of the ulcers (*OR* = 0.029, *p* = 0.001, 95% CI = 0.004–0.231), no predictor of *S. aureus*, MRSA, or polymicrobial ulcer infection was identified. Conclusions: The prevalence of *S. aureus* and MRSA infection of the diabetic foot ulcers were high, but lower than those of the predominant infector, coagulase-negative Staphylococci and the next highest infecting agent, *E. coli*. Diabetic foot ulcers’ infection with coagulase-negative Staphylococci protected against their infection with *S. aureus*. The prevalence of multidrug resistance was high, highlighting the need to further intensify antimicrobial stewardship programmes.

## 1. Introduction

*Staphylococcus aureus* (*S. aureus*) is a human-cum-animal pathogen that is predominantly harboured in the anterior nares of humans [1]. As a pathogen, it is capable of causing infections of the blood, endocardium, meninges, and the bone, and is the key infector of diabetic foot ulcers (DFUs) [2,3,4]. It has a rich arsenal of virulence determinants that mediate its success as a pathogen, such as adhesins that facilitate colonisation and entry of host cells [5,6,7]. Some others include the formation of biofilms, possession of a polysaccharide capsule, and expression of enzymes like coagulase, staphylokinase, hyaluronidase, and lipase, a composite of which protect the organism from destruction by both the immune system and antibiotics, giving it the capacity to persist in its host and invade tissues [8,9,10,11,12,13,14,15]. The clinical significance of the pathogen has been exacerbated by the emergence and rapid spread of multidrug resistance among its strains. One key multidrug-resistant *S. aureus* variant is methicillin-resistant *S. aureus* (MRSA), and its prevalence in foot ulcers has increased over the years, slowing down their healing rates and commonly resulting in amputations [4,16,17,18]. MRSA is resistant to major antibiotic groups in routine use [19,20], and its infections are synchronous with extended hospital stays as well as increased healthcare costs which could be as high as 44 million Euros [21,22,23].

About 463 million of the global population of adults are affected by diabetes, and this figure is expected to increase by at least 1.5 folds by the year 2045 [24]. Low- and middle-income countries have a disproportionate share of the diabetes burden, harbouring approximately 80% of the world’s diabetes population [24]. Africa, which is a predominantly low-income region, is expected to have about 49 million of its residents developing diabetes by 2045, a 17 million increase over the 2019 burden [24]. Moreover, most diabetes diagnoses in the region coincide with times of development of diabetic complications, such as DFUs [25]. Globally, an estimated 15–25% of diabetics develop foot ulcers, infections of which predominantly result in lower-extremity amputation and independently predict mortality [26,27,28].

It is estimated that each year, US$8659 is needed to treat each DFU patient [29], and this could overwhelm the already poorly-resourced healthcare systems of low-income regions. In order to improve the management and prognoses of diabetic foot ulcers, as well as reduce their economic burden, it is important to continuously monitor the range of organisms infecting these ulcers, as well as their antimicrobial resistance patterns. However, such surveillance studies are limited in resource-poor settings. To help fill this knowledge gap, this study investigated the spectrum of bacteria infecting the ulcers of individuals with diabetes at the Korle Bu Teaching Hospital in Accra, Ghana, focusing on *S. aureus* and MRSA, with respect to their prevalence, factors predisposing to their infection of the ulcers, and antimicrobial resistance patterns.

## 2. Results

### 2.1. Sociodemographic and Clinical Features of the Participants

In total, one hundred (100) individuals with active DFU participated in the study. Their sociodemographic data are presented in Table 1. The majority of the participants were more than sixty years old (57.0%), were females (54.0%), lived in self-contained apartments (57.0%), resided with 5 to 10 persons in their households (51.0%), did not have a health worker as part of their households (68.0%), and often washed their hands with soap (77.0%).

As regards the participants’ clinical features, a higher proportion had a history of hospitalisation in the past year (55%), lacked a history of pneumonia (95%), tuberculosis (97%), and surgery (66%), and reported that they did not practice self-medication (53%). Details of the clinical features are presented in Table 2.

### 2.2. Bacteria Infecting the Diabetic Foot Ulcers and Predictors of S. aureus and MRSA Foot Ulcer Infection

In total, 96% (*n* = 96) of the participants had their ulcers infected—32.3% (*n* = 31) of these had their ulcers infected with one bacterium, 47.9% (*n* = 46) with two bacteria, 18.8% (*n* = 18) with three bacteria, and 1.0% (*n* = 1) with four bacteria (coagulase-negative Staphylococci [CoNS], *Pseudomonas* spp., *Enterococcus* spp., and *Proteus mirabilis*). Consequently, the proportion of participants with polymicrobial ulcer infection was 65% (*n* = 65). Four of the participants had no evidence of bacterial infection of their ulcers.

As observed in Table 3, the predominant bacterium isolated from the foot ulcers of the participants was CoNS (54.0%). *Escherichia coli* recorded the second highest prevalence (24.0%), followed by *S. aureus* and *Pseudomonas* spp., each of which recorded a prevalence of 19% (*n* = 19). The prevalence of MRSA in the ulcers was 6%, and reflected a proportion of 31.6% of the participants who had *S. aureus* foot ulcer infections.

The multivariate analysis revealed that none of the participants’ sociodemographic and clinical features emerged as a significant predictor of MRSA infection of the foot ulcers, nor of polymicrobial infection of the ulcers. The only identified predictor was DFU infection with CoNS, and it was protective of *S. aureus* infection of the diabetic foot ulcers (*OR* = 0.029, *p* = 0.001, 95% CI = 0.004–0.231).

### 2.3. Antimicrobial Resistance Patterns of the S. aureus Isolates

The resistance rates of *S. aureus* decreased across the tested antimicrobials as follows: penicillin (100%, *n* = 19), tetracycline (47.4%, *n* = 9), cotrimoxazole (42.1%, *n* = 8), cefoxitin (31.6%, *n* = 6), erythromycin and clindamycin (26.3% each, *n* = 5), norfloxacin and gentamicin (15.8% each, *n* = 3), rifampicin (10.5%, *n* = 2), linezolid (5.3%, *n* = 1), and fusidic acid (0.0%, *n* = 0). The proportion of multidrug resistance (defined as resistance to three or more antimicrobial classes) [30] among the *S. aureus* isolates was 47.4% (*n* = 9).

## 3. Discussion

This study aimed to investigate the spectrum of bacteria infecting the ulcers of individuals with diabetes at the Korle Bu Teaching Hospital in Accra, Ghana, focusing on *S. aureus* and MRSA with respect to their prevalence, predictors of their infection of the ulcers, and antimicrobial resistance patterns. It fills an important knowledge gap in resource-poor settings where such studies are limited.

The study found a high DFU infection rate among the participants, and identified a diverse spectrum of Gram-positive and Gram-negative bacteria as the infecting agents, with a high prevalence of polymicrobial infections, which aligns well with previous reports that DFUs are predominantly polymicrobial [31,32]. The observation is, however, quite unsettling, as a high bacterial presence in ulcers is associated with a reduced ulcer healing rate [33]. Furthermore, it increases the propensity for biofilm formation by infecting bacteria, which complicates treatment and increases ulcer chronicity [34,35,36].

That CoNS are normal flora of the skin may have influenced their high prevalence in the foot ulcers of the participants. In fact, they are typically viewed as contaminants in clinical samples like swabs rather than pathogenic, unless the samples originated from deep tissues [37,38]. Even so, it is difficult to conclude that they were mere contaminants in this study, especially as the distinction between contaminant and pathogenic CoNS is still blur [39]. Besides, several studies have demonstrated CoNS to have comparable pathogenicity to *S. aureus*, as well as an increased drug resistance tendency [40,41,42,43,44]. Moreover, in this study, their presence was found to be protective of *S. aureus* infection of the foot ulcers. It is noteworthy that this observation aligns with the reported inverse relationship between *S. aureus* and CoNS, which has been attributed to the production of the *S. aureus*-cidal autoinducing peptide by CoNS [30,45,46].

*E. coli* was the second-highest bacterium isolated from the ulcers of the study participants. A similar finding was reported in a study conducted at the Komfo Anokye Teaching Hospital, another tertiary care hospital in Ghana, in which *E. coli* (24%) was second to *Proteus* species (31%) with regard to the proportion of microorganisms infecting the ulcers [47]. It is noted, however, that Brenyah et al.’s [47] study involved only 27 participants. The observation that *E. coli* was the second-highest bacterium isolated from the ulcers along with the 19% prevalence of *Pseudomonas* spp., agree with studies that have identified Gram-negative bacteria to be the predominant infective agents in foot ulcers [32,48,49].

Contrary to what has been reported in some other studies, such as those of Cervantes-García et al. [50] and Mutonga et al. [51] conducted in Mexico and Kenya, respectively, *S. aureus* was not the predominant bacterium isolated from the ulcers of the participants of the current study. In the study of Cervantes-García et al. [50], the prevalence of *S. aureus* was 42%, followed by *E*. *coli* (36%) and CoNS (25%), and in that of Mutonga et al. [51], the prevalence of *S. aureus* was 16%, followed by *E. coli* (15%), *P. mirabilis* (11%), and *P. aeruginosa* and *K. pneumoniae* (7% each). That notwithstanding, the *S. aureus* prevalence of 19% recorded in this study is still high. Moreover, that MRSA prevalence was 6% and accounted for 31.6% of these *S. aureus* isolates is a cause for concern, given the high clinical significance of the pathogen. It is noted, though, that this prevalence is below the 15% to 30% prevalence usually attributed to MRSA in DFUs [52] and may be a reflection of the low MRSA prevalence in the country [30,53,54,55,56,57,58].

The highest proportion of *S. aureus* resistance was recorded against penicillin (100%). Previous studies in the country have also reported similar rates for the organism [30,53,54,56,57,58], and this may be due to the wide usage of the antibiotic. These studies additionally recorded similar antimicrobial resistance rates for the other antimicrobials investigated [30,53,54,56,57,58]. Of particular interest is the high resistance displayed against cotrimoxazole in the current study, given its usage in prophylaxis. This rate of resistance against the antibiotic warrants its re-evaluation as a prophylactic agent and may add to the several lines of evidence that point to a need to further restrict prophylaxis administration, owing to its potential contribution to the ever-increasing antimicrobial resistance menace [59,60,61,62,63]. Moreover, the high proportion of MDR *S. aureus* accentuates the need for more rigorous antimicrobial stewardship programmes.

This study was limited by a few factors. First, the samples were obtained by swabbing the ulcers, and this technique has been reported in some studies to have a lower sensitivity [38,64]. Second, the bacterial detection was solely based on cultural methods, without including microbiome analysis, and this may have limited the range of bacteria detected. Moreover, data on the grade and duration of ulcers were absent.

It is concluded that the prevalence of *S. aureus* and MRSA infection of the diabetic foot ulcers were high, but lower than those of the predominant infector, coagulase-negative Staphylococci and the next highest infecting agent, *E. coli*. Diabetic foot ulcers’ infection with coagulase-negative Staphylococci protected against their infection with *S. aureus*. The prevalence of multidrug resistance was high, highlighting the need to further intensify antimicrobial stewardship programmes.

## 4. Materials and Methods

### 4.1. Study Site, Design, and Sampling

The Ulcer Clinic of the Department of Surgery, Korle Bu Teaching Hospital (KBTH), served as the site for conducting this cross-sectional study. The clinic attends to the wounds of individuals with diabetes who are received at the National Diabetes Management and Research Centre (NDMRC), KBTH, the largest diabetes centre in Ghana (which has over 5000 registered patients), as well as those without diabetes. The clinic attends to about 35 persons on a daily basis, from Monday to Saturday.

In total, one hundred (100) individuals with diabetic foot ulcers were consecutively recruited between January and June 2020. All 13 to 80 year old DFU patients, who upon physical examination, were found to have unhealed ulcers were deemed as active DFU patients, and were eligible for inclusion in the study. Confirmation of their diabetes status was done by reviewing their folders. Those who were unwilling to participate, had concurrent severe disease or had received antimicrobial treatment (including application of herbs, honey, or any other form of antimicrobial agent on their ulcers) two weeks prior to sampling were excluded from the study.

Informed consent was obtained from each participant. A structured questionnaire was used to collect sociodemographic and clinical data of the participants. Subsequently, for each participant, the wound area was rinsed with sterile normal saline, followed by swabbing of the centre of the wound with a sterile cotton swab. Each swab sample was placed into a uniquely tagged, sterile, 1 mL skim milk-tryptone-glucose-glycerin (STGG)-contained vial. These were transported to the Department of Medical Microbiology, University of Ghana Medical School, within four hours, for laboratory processing, which entailed initial vortexing for two minutes and subsequent storage at −80 °C, until needed.

### 4.2. Laboratory Analysis

Slight modifications of the steps described by Donkor et al. [30] guided the laboratory analyses—identification of *S. aureus*, MRSA, and other bacteria, antimicrobial susceptibility testing of *S. aureus*, and molecular investigations. Mannitol salt agar (Oxoid, Basingstoke, Hants, UK), MacConkey agar (Oxoid, Basingstoke, Hants, UK), 5% sheep blood agar (Oxoid, Basingstoke, Hants, UK), and chocolate agar (Oxoid, Basingstoke, Hants, UK) were used in culturing the samples following their enrichment in tryptic soy broth (Oxoid, Basingstoke, Hants, UK). The biochemical analyses conducted included catalase, tube coagulase, oxidase, indole, triple sugar iron, and citrate tests (Becton and Dickinson^®^; Heidelberg, Germany). Staphylococcal isolates that were coagulase-negative and -positive were respectively identified as coagulase-negative Staphylococci (CoNS) and *S. aureus*. In accordance with the Clinical and Laboratory Standards Institute (CLSI M100-S30) guidelines [65], susceptibilities of the *S. aureus* isolates were tested against norfloxacin (10 µg), erythromycin (15 µg), cotrimoxazole (1.25 µg trimethoprim + 23.75 µg sulphamethoxazole), clindamycin (2 µg), tetracycline (30 µg), rifampicin (5 µg), linezolid (10 µg), fusidic acid (10 µg), gentamicin (10 µg), penicillin (10 units), and cefoxitin (30 µg) (Oxoid, Basingstoke, Hants, UK). A summary of the interpretive categories (Table 4) and zone breakpoints for the antibiotics is presented in Table 1. The control strain used was *S. aureus* ATCC 25923 (Becton and Dickinson^®^; Heidelberg, Germany). Confirmation of cefoxitin-resistant *S. aureus* as *S. aureus* and MRSA was carried out via polymerase chain reaction (PCR) amplification of the *nuc*A and *mec*A genes, respectively.

Following the instructions of the manufacturer, the Zymo Research extraction kit (Zymo Research Corp., Irvine, CA, USA) was used to extract genomic DNA from overnight lysogenic broth cultures of the presumptive MRSA isolates (including an in-house positive control 16S ribosomal RNA sequencing-confirmed MRSA isolate) during the molecular analyses. For quality control purposes, the extracted DNA (5 µL volume) of each isolate was mixed with 2 µL of bromophenol blue gel loading buffer, followed by size separation using a 1.2% (w/v) agarose gel electrophoresis. The bands on the resultant gel were viewed under ultraviolet light, and each DNA sample was used as a template in the PCR of the *mecA* and *nucA* genes.

### 4.3. Data Analysis

The data analysis was performed using STATA 14 (Strata Corp, College Station, TX, USA). Summary of the sociodemographic, clinical, and antimicrobial resistance data was done using descriptive statistics. Using an alpha level of 0.05, univariate and multivariate analyses (including odds ratios and 95% confidence intervals) were employed in determining risk factors for *S. aureus*, MRSA, and polymicrobial infection of the ulcers.

### 4.4. Ethical Approval

The approval for conducting this study was given by the Institutional Review Board of the Korle Bu Teaching Hospital (Unique Identification: KBTH-STC/IRB/000144/2019) and the Ethical and Protocol Review Committee of the College of Health Sciences, University of Ghana (Unique Identification: “CHS-Et/M.3–9.16/2019-2020”).

## Figures and Tables

**Table 1:** Demographic and household characteristics of the study participants.

Demographic and Household Characteristics	Number	%
*Age (in years)*		
14–19	1	1.0
20–29	0	0.0
30–60	42	42.0
>60	57	57.0
*Gender*		
Male	46	46.0
Female	54	54.0
*Type of residence*		
Self-contained	57	57.0
Compound	43	43.0
*Number of individuals in household*		
<5	44	44.0
5–10 persons	51	51.0
11–20 persons	5	5.0
*Presence of health worker in household*		
Yes	32	32.0
No	68	68.0
*Hand washing with soap*		
Rarely	23	23.0
Often	77	77.0

Age $\bar{(X\;},\;SD)$ = 61.76, 14.70 years; Number of individuals in household $\bar{(X},\;SD)$ = 5.44, 2.87 persons.

**Table 2:** Clinical characteristics of the study participants.

Clinical Characteristics	Number	%
*Self-reported self-medication*		
Yes	47	47.0
No	53	53.0
*History of hospitalisation in the past year*		
Yes	55	55.0
No	45	45.0
*History of pneumonia*		
Yes	5	5.0
No	95	95.0
*History of tuberculosis*		
Yes	3	3.0
No	97	97.0
*History of surgery*		
Yes	34	34.0
No	66	66.0

Number of hospitalisations $\bar{(X},\;SD)$ = 0.99, 1.22.

**Table 3:** Bacteria isolated from the diabetic foot ulcers.

Bacterium	Number	Prevalence (%)
*Staphylococcus aureus*	19	19.0
MRSA	6	6.0
CoNS	54	54.0
*Escherichia coli*	24	24.0
*Streptococcus* spp.	1	1.0
*Enterococcus* spp.	6	6.0
*Klebsiella* spp.	2	2.0
*Klebsiella pneumoniae*	5	5.0
*Klebsiella oxytoca*	11	11.0
*Citrobacter koseri*	12	12.0
*Enterobacter* spp.	4	4.0
*Morganella morgana*	12	12.0
*Pseudomonas* spp.	19	19.0
*Proteus vulgaris*	8	8.0
*Proteus mirabilis*	4	4.0

**Table 4:** A summary of the interpretive categories and zone breakpoints.

Antibiotic	Interpretive Categories and Zone Diameter Breakpoints (mm)		
	Susceptible	Intermediate	Resistant
Penicillin	≥29	–	≤28
Tetracyline	≥19	15–18	≤14
Cotrimoxazole	≥16	11–15	≤10
Cefoxitin	≥22	–	≤21
Erythromycin	≥23	14–22	≤13
Clindamycin	≥21	15–20	≤14
Norfloxacin	≥17	13–16	≤12
Gentamicin	≥15	13–14	≤12
Rifampicin	≥20	17–19	≤16
Linezolid	≥21	–	≤20
Fusidic acid	≥33	24–32	≤23

## Data Availability

The data presented in this study are available upon reasonable request from the corresponding author via esampane-donkor@ug.edu.gh.
